# Development of a Konjac-Based Surgical Training Model Exhibiting Energy Device Applicability and Tactile Similarity to Animal Tissue

**DOI:** 10.7759/cureus.91506

**Published:** 2025-09-02

**Authors:** Gaku Morimoto, Yuma Endo, Sosaku Ichikawa

**Affiliations:** 1 Technical Department, Kotobuki Medical Inc., Yashio, JPN; 2 Institute of Life and Environmental Sciences, University of Tsukuba, Tsukuba, JPN

**Keywords:** dissection force, plant-based material, surgical simulation, surgical training model, tactile similarity

## Abstract

Introduction: The use of simulation models in surgical training allows trainees to improve their in-hospital performance. While there are many types of models to simulate human tissue, including animal meat, polyvinyl alcohol, and silicon, each faces some challenges in terms of preservability and energy device applicability. We propose that konjac glucomannan (KGM) is a realistic alternative that can provide trainees with a texture closely resembling that of human tissue. This study was undertaken to describe the similarities between KGM gel and animal meat, as well as to investigate the effect that specific preparation methods can have on KGM gel's texture.

Methods: Three types of konjac models (KMs) and animal meat models, including porcine back muscle, porcine liver, and chicken muscle, were prepared. The strength and strain of each model were tested in a way that mimics blunt dissection. The microstructure of KM was also observed via scanning electron microscopy.

Results: The texture analysis revealed that the strength and strain of the KMs are comparable to those of animal meat. We also found that the additional steps of freezing and moisture removal during the preparation process for the KM preparation have a significant impact on the strength and the strain of the models.

Conclusion: KMs that are prepared in an appropriate way have great potential for use in surgical training. We believe that KM should be considered a valuable model for medical trainees to experience more realistic surgical training.

## Introduction

Minimally invasive surgical technologies, such as laparoscopy and robotic-assisted surgery, have increasingly become standard practice among medical professionals. Studies have shown that in order for surgeons to adopt such technologies and continue improving patient outcomes, they should practice basic surgical procedures (e.g., blunt dissection with forceps, suturing, and cutting with energy devices) [1,2]. Trainees often use animals or animal meat due to the anatomical or tactile reality of such models [3,4]. However, there are also several disadvantages of using meat-based models, one of which is that they cannot be preserved easily. Apart from a short shelf life, there are also risks associated with hazardous compounds produced from animal meat when using electrocautery in training [5] and some restrictions on use related to religious and ethical perspectives [6]. Therefore, alternative synthetic materials such as polyvinyl alcohol (PVA) and silicone have been developed to be used in surgical training [7]. Several studies have compared biological tissue with synthetic materials. For example, Gul et al. reported that synthetic models made from silicone, cotton, and dyes can provide a realistic texture to be used in a simulation of nerve microsurgery [8]. Also, de Jong et al. investigated that a 4 wt% PVA hydrogel that undergoes two freeze-thaw cycles of freezing at -19°C can mimic the liver tissue [9].

By contrast, KOTOBUKI Medical Inc. developed a model called Versatile Training Tissue (VTT), which is made from konjac glucomannan (KGM). KGM is a dietary fiber hydrocolloidal polysaccharide isolated from the tubers of *Amorphophallus konjac* [10]. KGM is widely used in Japan's food industry as a gelation agent due to its unique colloidal properties of effective viscosity enhancement and thermal-irreversible gelling and its unique texture when used in food [10].

The KGM model has significant potential for use in surgical training due to its viability with electrocautery and safety during use [11-14]. Morimoto et al. reported that the concentration of organic compounds (e.g., acrolein) in smoke from a KGM model is lower than in smoke from PVA gel and porcine muscle when using an electrocautery [11]. Nishio et al. showed that a KGM model with a fluorescent agent enables learning of NIR fluorescence imaging and supports the clinical simulation of fluorescence-guided cancer surgery [12]. However, few studies describe the tactile similarity between models such as the KGM model and animal tissue, particularly with respect to clinically relevant parameters such as blunt dissection stress. Evaluating the elasticity and flexibility of the KGM model under conditions simulating actual surgical procedures, such as blunt dissection, and comparing these values with those of animal tissue is important for demonstrating its effectiveness as a training model. Additionally, it has been recognized that the method of preparation of KGM gels influences their physical properties from the viewpoint of polymer science [15]. However, no systematic knowledge has been reported regarding the relationship between differences in preparation methods and the resulting physical properties in relation to their utilization for surgical training purposes.

In this study, we focused on konjac models (KMs), which are used for surgical training and are made of KGM. As these models have a similar texture to animal tissue, such as porcine back muscle, we expected that they would exhibit quantitatively similar strength and strain to animal models. We also report on the applicability of KMs being used with an electrocautery. The purpose of this study was to compare the tactile properties of KM with animal meat, as well as to describe the effect the preparation methods for KM had on its tactile properties.

## Materials and methods

Materials

Konjac flour with a specific particle size was obtained by using a 30-mesh sieve (Moteki Foods Engineering Co., Ltd., Shimonita, Japan) and was used as a raw material to make KM. Scallop shell-derived calcium hydroxide (Moteki Foods Engineering Co., Ltd.) was also obtained. Commercial specimens of porcine back muscle, porcine liver, and chicken muscle were purchased. Sodium chloride (Hayashi Junyaku Co., Osaka, Japan) was obtained and used to provide prepared models with conductivity for electrocautery.

Preparation of KMs

**Figure 1:**
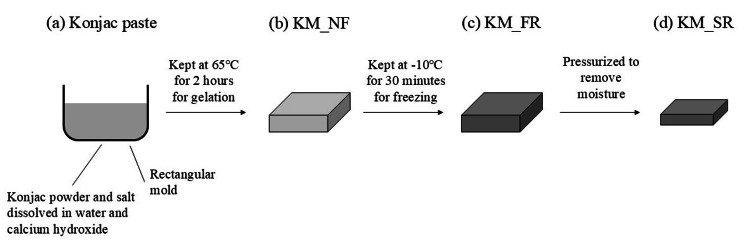
A schematic diagram of the preparation of konjac models (KM): (a) konjac paste; (b) konjac gel obtained by heating konjac paste at 65°C for two hours (KM_NF); (c) KM_NF frozen and thawed (KM_FR); and (d) KM_FR desiccated (KM_SR).

The experimental setup for creating KMs is shown in Figure 1. First, 10 g of konjac powder and 2 g of sodium chloride were dissolved in 600 mL of water (Nihon Millipore K.K., Tokyo, Japan). Then, 80 mL of calcium hydroxide (1.5 wt%) was added to the mixture to create konjac paste (KM paste, Figure 1a). We then created a non-frozen KM (KM_NF, Figure 1b) by heating the paste in a rectangular mold for two hours at 65°C. Following this, we made a frozen KM (KM_FR, Figure 1c) by keeping the KM_NF in a low-temperature environment (-10°C) for 10 hours and then thawing it by keeping it at 15 °C for 10 hours. Finally, a weight was placed on top of a KM_FR, and a load of 8 kg was applied. The pressure applied was 28 kilopascals. This pressure was applied for at least five hours to create the syneresis KM (KM_SR, Figure 1d). This was done to remove some of the moisture from the model. The degree of syneresis for each gel was controlled by adjusting the duration of pressure applied to the gel. We measured the mass of the KM_FR at various time points before reaching a certain syneresis ratio.

**Figure 2:**
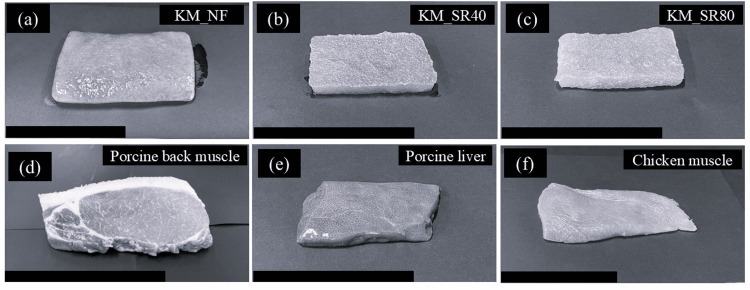
Photographs of tested models (scale bar = 10 cm): (a) konjac gel obtained by heating konjac paste at 65°C for at least two hours (KM_NF); (b) KM_NF frozen, thawed, and desiccated at the syneresis ratio of 40% (KM_SR40); (c) KM_NF frozen, thawed, and desiccated at the syneresis ratio of 80% (KM_SR80); (d) a slice of porcine back muscle; (e) a slice of porcine liver; (f) a slice of chicken muscle.

In this study, we adjusted the syneresis ratio of two gels to approximately 40% and 80%, and it took approximately 10 hours to reach 80% of the syneresis ratio. These gels are labeled KM_NF, KM_SR40, and KM_SR80 in Figures 2a, 2b, 2c, respectively. In this study, at least five of each of those labeled samples were prepared. Furthermore, KM_FM with syneresis ratios between 40% and 80% was also prepared by adjusting the dehydration time to less than 10 hours. These gels with randomized syneresis ratios were utilized for generating a scatter plot to discuss the relationship between the syneresis ratio and the textural property. The syneresis ratio is determined by the following formula: $\text{Syneresis ratio (\%)} = \frac{(X - Y)}{X} \times 100$, where X is the weight of a KM_NF and Y is the weight of a KM_SR.

Texture analysis of prepared models

**Figure 3:**
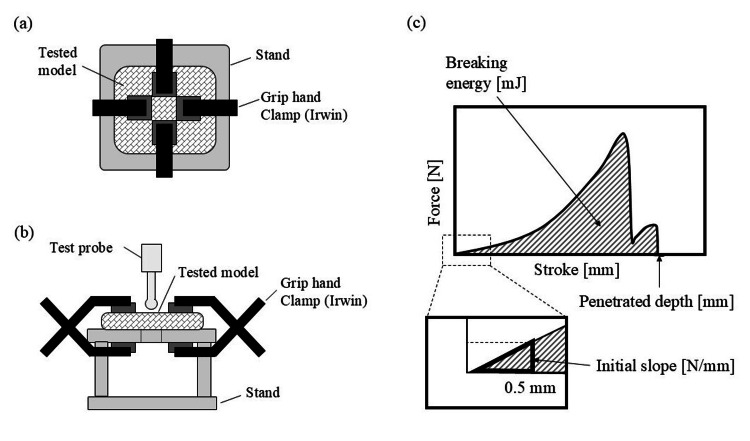
A schematic diagram of the procedures for the texture analysis of prepared models: (a) top view of the measurement, (b) side view of the measurement, (c) evaluated parameters.

Although tensile strength testing has been widely investigated for comparing the texture of training models with that of animal tissues [16], we adopted a different approach in this study. Specifically, we compared the textural properties of the KMs and animal meat models under conditions that more closely simulate actual surgical procedures, such as blunt dissection (the separation of tissue using the tip of a non-sharp instrument). To this end, we compared the textual properties of KMs and animal meat using a penetration test. We considered this approach more appropriate for evaluating the practical applicability of the training model. The tests were performed at room temperature using a texture analyzer (EZ-SX, SHIMADZU Corporation, Kyoto, Japan). In this test, a 5 mm diameter probe penetrated each model at a rate of 1 mm/s. The diagrams for the penetration test are shown in Figures 3a, 3b. The force (Newtons) and the distance (millimeters) when the model was completely penetrated were also recorded. This penetration test was performed once for each single sample and repeated at least five times for each labeled sample under each preparation method. In this study, two parameters were evaluated: breaking energy (megajoules) and strain (-). The tactile features of each model were compared based on these two factors. As shown in Figure 3c, breaking energy represents the area surrounded by the force-stroke curve and the horizontal axis. The strain measurement was obtained by dividing the penetration depth of each model by the thickness of the model.

Microstructure observation of KM using scanning electron microscopy (SEM)

Given that it was expected that the preparation methods for KM would affect its tactile properties due to changes in the gel's microstructure, SEM observations were performed to observe the gel's microstructure and support this phenomenon. SEM images were observed with a scanning electron microscope (Miniscope TM-1000, Hitachi High-Technologies, Tokyo, Japan). Prior to the observation, each sample was coated with Nanosuit® (NanoSuit Inc., Hamamatsu, Japan) [17]. NanoSuit® forms a 50-100 nm thin polymer membrane on the surface of the sample when irradiated with electron beams, which inhibits the release of water in the sample. Therefore, we considered that the KMs could be observed in a wet form.

Confirmation of applicability for using electrocautery

To confirm the applicability of using electrocautery, an electrocautery unit (Martin ME 401, Martin Medizin-Technik, Tuttlingen, Germany) was used to cut the KM_SR80 with the "cut" mode set at 30 W.

## Results

**Figure 4:**
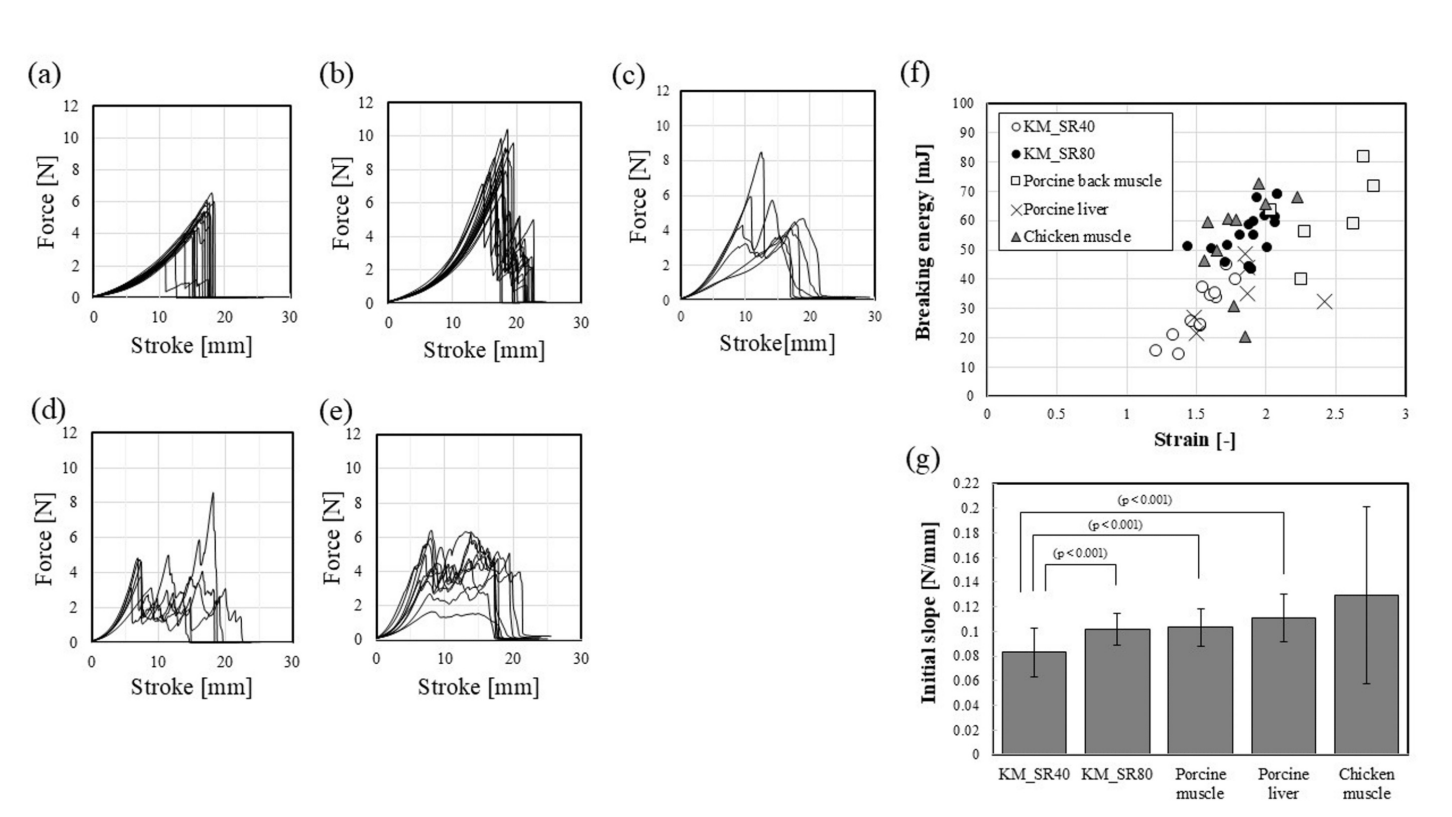
The result of the texture analysis of prepared models The force-stroke curve (a) KM_NF (konjac gel obtained by heating konjac paste at 65°C for two hours) frozen, thawed, and desiccated at the syneresis ratio of 40% (KM_SR40), (b) KM_NF (konjac gel obtained by heating konjac paste at 65°C for at least two hours) frozen, thawed, and desiccated at the syneresis ratio of 80% (KM_SR80), (c) porcine back muscle, (d) porcine liver, (e) chicken muscle (Y-axis is for the force and X-axis is for the stroke of the probe), (f) the scatter plots of breaking energy and strain of tested models, and (g) initial slopes for each model (p-values above brackets indicate the significance of model-related changes in the initial slopes. All p-values are based on paired t-tests corrected for multiple comparisons. Error bars are the standard error of the mean.

Figures 4a-e show the force-stroke curve of each model. KM_NF did not have sufficient gel strength to be tested, and no data were obtained. The scatter plot for comparing the correlation between the breaking energy and the strain of each model is described in Figure 4f. In addition, initial slopes for each model are shown in Figure 4g. KM_SR80 exhibited more similarity to animal meat than KM_SR40 in terms of the breaking energy, the strain, and the initial slope.

**Figure 5:**
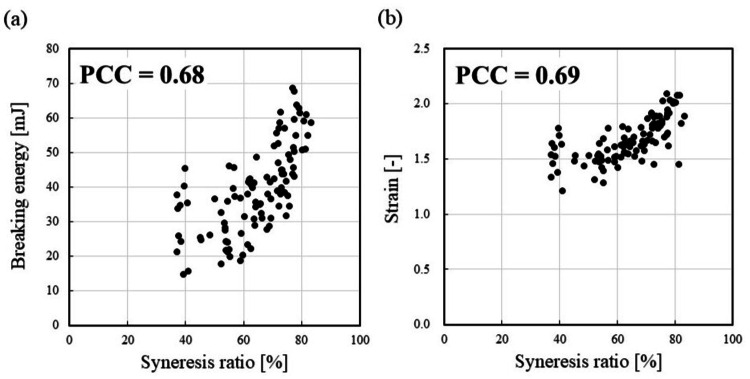
Relationship between the syneresis ratio of the konjac model and its strength, and correlation coefficient according to Pearson (PCC) between the x-axis and y-axis: (a) the scatter plot of breaking energy and syneresis ratio of konjac models; (b) the scatter plot of strain and syneresis ratio of konjac models.

Figure 5 contains correlation graphs. In Figure 4, the breaking energies of KM_SR with specific syneresis ratios were compared with those of other prepared models. In contrast, Figure 5 aimed to experimentally elucidate the effect of syneresis ratio on breaking energy and strain by preparing KM_SRs with various syneresis ratios at random and measuring their respective strengths. The correlation between breaking energy and syneresis ratio is shown in Figure 5a. The correlation between strain and syneresis ratio is shown in Figure 5b. The correlation coefficients according to Pearson (PCC) were 0.68 for the relationship between breaking energy and the syneresis ratio of KM and 0.69 for the relationship between strain and the syneresis ratio of KM.

**Figure 6:**
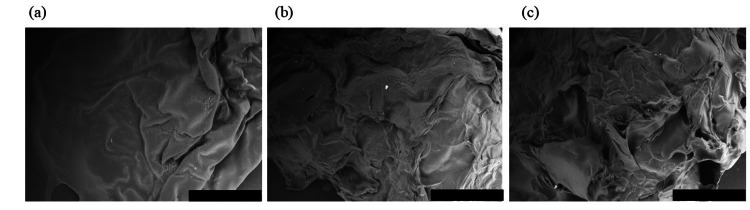
SEM images of konjac models (scale bar = 1 mm): (a) konjac gel obtained by heating konjac paste at 65°C for at least two hours (KM_NF); (b) KM_NF frozen, thawed, and desiccated at the syneresis ratio of 40% (KM_SR40); and (c) KM_NF frozen, thawed, and desiccated at the syneresis ratio of 80% (KM_SR80).

The obtained SEM images of the KM_NF, KM_SR40, and KM_SR80 are shown in Figures 6a, 6b, 6c, respectively.

The KM_SR80 was successfully cut with an electrocautery device (Video 1).

**Video 1:** A video of a KM_NF (konjac gel obtained by heating konjac paste at 65°C for at least two hours) frozen, thawed, and desiccated at the syneresis ratio of 80% (KM_SR80), being cut with an electrocautery.

## Discussion

We carried out comparative strength tests using the various models and found that the breaking strength, the strain, and the initial slope of the KM_SR80 were more similar to porcine back muscle, porcine liver, and chicken meat than the KM_SR40. Given that Bastos and Silva described that it is important to consider the tactile properties of the model when trainees learn basic surgical skills such as suturing [18,19], it is desirable to choose the KM_SR80 in such a situation based on the results of this study. Ideally, a model is needed to tear when the trainee makes a wrong movement, like applying excessive force when suturing [19]. Therefore, a training model should possess a material strength that is not too strong nor too weak for operations such as suturing. The similarity of the initial slope of the KM_SR80 to that of the animal meat (Figure 4g) possibly implies that the stiffness of the tissue is realistic and beneficial for a training that involves palpation.

Previous studies have indicated that training with models possessing texture properties similar to those of biological tissues is practically useful [20]. Therefore, it can be considered that there is a strong association between quantitative texture measurements and surgical applicability. With respect to blunt dissection, although the actual practice is diverse and cannot be fully reproduced by texture measurements alone, measuring the breaking energy and strain at the point of model failure in penetration tests provides valuable information. In particular, such measurements are considered effective for evaluating the sense of the force feedback experienced when tissues are separated during blunt dissection.

The animal model showed chaotic behavior, with force value fluctuating up and down after the first failure, while the KM also showed force fluctuating up and down, but more significantly in the animal model (Figures 4a-e). This is thought to be due to the fact that the animal model contains fibers and fats and thus is composed of heterogeneous tissues compared to KM.

The breaking strength of the KMs varied. By comparing Figure 4a with Figure 4b, it is clear that the breaking strength of both KM_SR40 and KM_SR80 was higher than that of the KM_NF, considering the KM_NF did not have sufficient strength to be tested. We posit that the increase in gel strength is attributed to the change in KGM's texture during the freeze-thaw process. Genevro et al. reported that the freeze-thaw process provided a significant improvement in the physical properties of KGM gels [15]. They explained that the reason for this was that water crystallization during freezing led to KGM networks changing into ordered structures, forming pores inside the gel after thawing. We also found that there are pores inside the KM_SR40 and the KM_SR80 (Figures 6b, 6c). On the other hand, the KM_NF's microstructure was found to be relatively smooth with fewer pores (Figure 6a). The pores were formed in the KM_SR40 and KM_SR80 following the processes of freezing, thawing, and desiccation. Therefore, we consider that gel strength improves as a result of the increased density of the gel network structures.

The PCCs were 0.68 for the breaking energy of KM, with its syneresis ratio, and 0.69 for the strain of KM, with its syneresis ratio, demonstrating strong relationships in both cases (Figure 5). We believe that the higher the syneresis ratio, the denser the gel network structure, which leads to a strong tactile property. We considered that this is due to syneresis occurring, which increases the weight per unit volume of the gel.

For example, suppose we have 100 g of konjac paste and prepare the KM_SR40 and KM_SR80 from the konjac paste. The weight of the KM_SR40 is 60 g, whereas it is 20 g in the case of the KM_SR80. The KM_SR80 has three times the weight per unit volume compared to the KM_SR40.

The KM_SR80 was viable for use with an electrocautery device, and the tissue responded in a realistic manner while being cut (Video 1), indicating that KMs are viable in a surgical training scenario using electric medical devices.

## Conclusions

The results of this study showed that due to their similarities to animal meat in terms of breaking strength, KM_SR80 is a suitable choice for practicing basic surgical procedures (e.g., blunt dissection with forceps, suturing, and experimental use of energy devices). Furthermore, the results indicated that the syneresis ratio of the KM_FR can be adjusted to simulate the material strength of biological tissues and organs, allowing trainees to practice procedures in a more effective manner.
